# Anomalous muscles and nerves in the hand of a 94-year-old cadaver—A case report

**DOI:** 10.1016/j.ijscr.2019.10.062

**Published:** 2019-10-31

**Authors:** H.L. Nation, S.Y. Jeong, S.W. Jeong, A.P. Occhialini

**Affiliations:** a Department of Cell Systems and Anatomy, University of Texas-Health at San Antonio, 7703 Floyd Curl Dr, San Antonio, TX 78229, United States; b Long School of Medicine, University of Texas-Health at San Antonio, 7703 Floyd Curl Dr, San Antonio, TX 78229, United States

**Keywords:** ADM, Abductor Digiti Minimi, DUN, Dorsal branch of the Ulnar Nerve, FDMB, Flexor Digiti Minimi Brevis, FDML, Flexor Digiti Minimi Longus, APB, Abductor Pollicis Brevis, CPDA, Common Palmar Digital Arteries, FCR, Flexor Carpi Radialis, FCU, Flexor Carpi Ulnaris, FDP, Flexor Digitorum Profundus, FDS, Flexor Digitorum Superficialis, MN, Median Nerve, PB, Palmaris Brevis, PCL, Palmar Carpal Ligament, PL, Palmaris Longus, RMN, Recurrent branch of the Median Nerve, SPA, Superficial Palmar Arch, SUN, Superficial branch of the Ulnar Nerve, UA, Ulnar Artery, UN, Ulnar Nerve, Case report, Flexor digiti minimi longus, Lumbrial, Anomalous, Berrettini anastomosis, Kaplan’s anastomosis

## Abstract

**Introduction:**

During an anatomical dissection of the distal upper extremities, numerous muscular and nervous anomalies were found in the forearm and hand of a 94-year-old cadaver. These anomalies are clinically relevant with regard to medical or surgical interventions.

**Presentation of case:**

The presence of a “flexor digiti minimi longus” muscle was observed passing through Guyon’s canal; to our knowledge this passageway has never been previously reported. An aberrant first lumbrical with three origins was noted. Additionally, numerous atypical nerves were found innervating the hand; the dorsal branch of the ulnar nerve contributed to cutaneous innervation of the palm of the hand (Kaplan’s anastomosis), the superficial ulnar nerve provided muscular innervation to the flexor digiti minimi brevis muscle, and two connections between the common palmar digital branches of the median and superficial ulnar nerves were observed (Berrettini anastomosis).

**Discussion:**

Here, we describe an extranumerary muscle associated with the hypothenar group of muscles. We also describe unusual origins of the first lumbrical muscle, and atypical cutaneous and muscular innervation to the palm of the hand.

**Conclusion:**

Clinically, understanding the existence of these anatomical variations may influence medical care or surgical procedures.

## Highlights

• The additional flexor muscle (Flexor Digiti Minimi Longus m.) passing through Guyon’s canal is the first to be described.
• The anomalous first lumbrical muscle with three origins is the first to be described.
• Berretini’s and Kaplan’s anastomosis was also noted.

## 1 Introduction

Compression of neurovascular structures within Guyon’s canal and the carpal tunnel result in neuropathic, vascular and muscular changes. While compression of these nerves are common, it is important for clinicians to be aware of anatomic variations that could be the cause of these compressions. In this article, the authors will describe anomalous variations of hypothenar and first lumbrical muscles that likely caused compression in Guyon’s canal and the carpal tunnel. Additional variations in innervation in the hand were also seen and may have confounded the clinical picture.

The hypothenar muscle group is usually comprised of three muscles: abductor digiti minimi (ADM), flexor digiti minimi brevis (FDMB) and opponens digiti minimi. Both ADM and FDMB insert into the proximal phalanx of digit 5. The relative position of these muscles in the hypothenar region determine the action these muscles have mechanically on the 5th digit.

The Encyclopedia of Human Anatomic Variation lists several variations for FDMB and ADM [1]. Published variations of FDMB include abnormal origins, having accessory slips from the hook of the hamate of flexor carpi radialis muscle, being absent, or being duplicated [1]. Published variations of ADM include having abnormal origins, being united with flexor digiti minimi, having one, two, or three muscle bellies, or being absent [1]. A flexor digiti minimi longus (FDML), an extra flexor of the 5th digit, has been reported in the past although rarely [2,3]. Anomalous FDML muscles have been described as originating from the ulnar tuberosity, the intracompartmental septum, or the tendon of palmaris longus. The tendon of the FDML may pass through the carpal tunnel, Guyon’s canal, or superficial to the flexor retinaculum [[2], [3], [4]]. The common denominator is that the muscle fibers run in such a way that it would function similarly to FDMB, to flex the 5th digit. To the best of our knowledge, there has not been any report of a muscle belly of the flexor digiti minimi longus that passes through Guyon’s canal.

The lumbrical muscles of the hand are four short muscles that flex the metacarpophalangeal joints and extend the interphalangeal joints. Each lumbrical originates from the tendon of flexor digitorum profundus, distal to the flexor retinaculum, and inserts on the radial side of the extensor expansion hood of each digit. The first and second lumbricals are unipennate having one origin from the tendon of flexor digitorum profundus. The incidence of reported variations of the lumbricals increases from lateral to medial with the first lumbrical having the least variability [5]. Reported variations of the first lumbrical include the location of the origin (some originating proximal to the flexor retinaculum) and the number of heads (some reported to be bipennate, having an accessory belly from the flexor pollicis longus or flexor digitorum superficialis) [1]. To our knowledge, there has been no report of the first lumbrical muscle being tripennate and originating from the three unique sources as described in this case study.

Previous literature has described anatomical variations in the cutaneous and muscular innervation to the hand. Anastomoses between the median nerve and ulnar nerve on the palm are relatively well published by many articles. The most commonly reported anastomoses are the Riche-Cannieu anastomosis (a connection between the deep branch of the ulnar nerve and recurrent branch of median nerve) and the Berretini anastomosis (connections between common digital nerves of ulnar nerve and median nerve in the palmar aspect of the hand) [6]. An anastomosis between the dorsal branch of the ulnar nerve (DUN) and the superficial branch of the ulnar nerve is a rarer finding called Kaplan’s anastomosis [7]. Variations of Kaplan’s anastomosis have also been reported [[8], [9], [10]]. The nerve anomalies reported in this study fit the classic description of Berretini’s anastomosis and Kaplan’s anastomosis. To our knowledge, the innervation of flexor digiti minimi brevis by branches of the superficial ulnar nerve, observed in this case study, have yet to be described. The work reported within this manuscript is in line with the SCARE criteria [11].

## 2 Case presentation

**Fig. 1:**
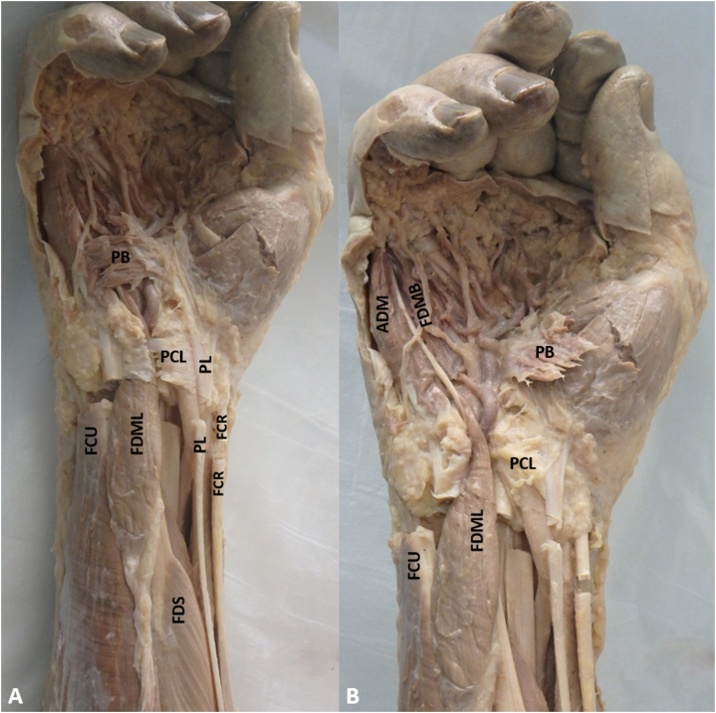
(A) FDML: Flexor Digiti Minimi Longus; PB: Palmaris Brevis; PL: Palmaris Longus; PCL: Palmar Carpal Ligament; FCU: Flexor Carpi Ulnaris; FCR: Flexor Carpi Radialis; FDS: Flexor Digitorum Superficialis; (B) FDMB: Flexor Digiti Minimi Brevis and ADM: Abductor Digiti Minimi muscles are visualized. PCL and PB are reflected to visualize the FDML muscle belly passing through Guyon’s canal.

During an anatomical dissection for medical students, several anomalous anatomical structures were noted on the right hand and forearm of an adult 94-year-old male cadaver. No anomalies were seen on the left upper extremity. Upon incising the antebrachial fascia, the belly of an anomalous muscle was observed to take origin from the deep surface of the distal two thirds of this fascia. The muscle belly passed through Guyon’s canal (Fig. 1A). The tendon of this muscle was traced to its insertion (Fig. 1B). The tendon of the anomalous muscle shared an insertion with the ADM and the FDMB (Fig. 1B). Due to this shared insertion with FDMB muscle, its length, and its direction of pull/action, the authors choose to name this accessory muscle “flexor digiti minimi longus” (FDML).

**Fig. 2:**
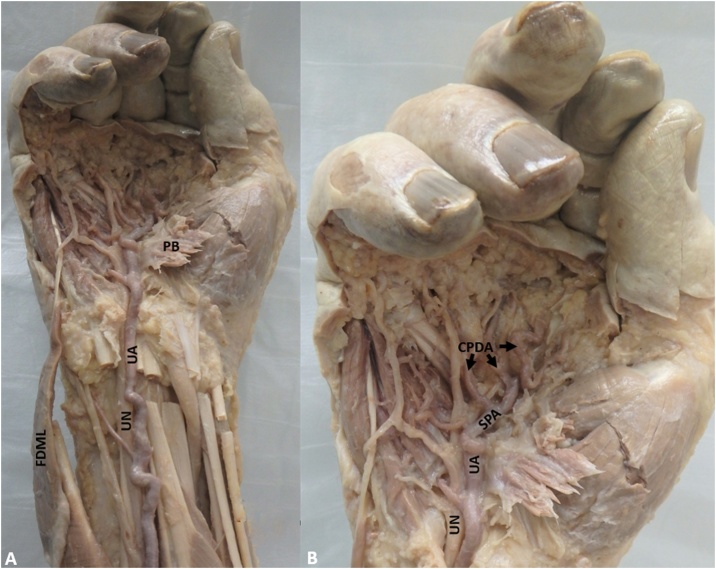
(A) FDML: Flexor Digiti Minimi Longus is reflected out of Guyon’s canal; PB: Palmaris Brevis (reflected); UA: Ulnar Artery; UN: Ulnar Nerve; (B) The tortuous SPA: Superficial Palmar Arch and CPDA: Common Palmar Digital Arteries are visible.

Deep to the FDML, the ulnar artery, superficial palmar arch and common digital arteries were all unusually tortuous (Fig. 2A and B) but with a normal pattern. The presentation in the above arteries is most likely a result of the adaptation to prolonged hypertension caused by compression in Guyon’s canal indicating the possibility of Guyon’s canal syndrome.

**Fig. 3:**
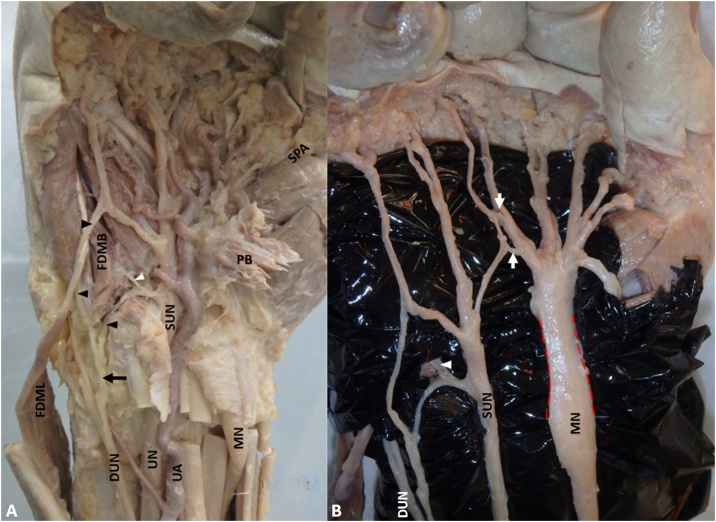
(A) FDML: Flexor Digiti Minimi Longus (reflected); FDMB: Flexor Digiti Minimi Brevis; MN: Median Nerve; UA: Ulnar Artery; UN: Ulnar Nerve; SUN: Superficial branch of the Ulnar Nerve; DUN: Dorsal branch of the Ulnar Nerve; Black Arrow: Aberrant branch of the dorsal branch of the ulnar nerve; Black Arrowheads: branches of the aberrant branch of the dorsal branch of the ulnar nerve on palmar surface of hand and anastomosing with the SUN; White Arrowhead: branch of the superficial ulnar nerve innervating FDMB; (B) White Arrows: Connection between SUN and MN, Red dashed lines: hourglass constriction of the MN.

Further dissection of the hand revealed numerous variations in the cutaneous and muscular innervation. Proximal to Guyon’s canal, before the dorsal branch of the ulnar nerve became cutaneous to the dorsal surface of the hand, it gave off an aberrant branch which then divided into two branches (Fig. 3A). The first branch provided cutaneous innervation to the palmar surface of the medial side of the 5th digit. The second branch joined the superficial ulnar nerve (Kaplan’s anastomosis). Branches of the superficial ulnar nerve (which is often considered a cutaneous nerve except its branch to the palmaris brevis muscle) were seen innervating the FDMB (Fig. 3A). To our knowledge, this is the first report of such a case.

With additional dissection, the transverse carpal ligament was reflected; synovial thickening of the flexor digitorum superficialis and profundus muscles was noted. The median nerve had narrowing within the carpal tunnel and widening again distally, indicating the possibility of carpal tunnel syndrome (Fig. 3B). Two aberrant anastomosis between the common palmar digital branches of the median and superficial ulnar nerves were also identified (Berretini anastomosis) (Fig. 3B).

**Fig. 4:**
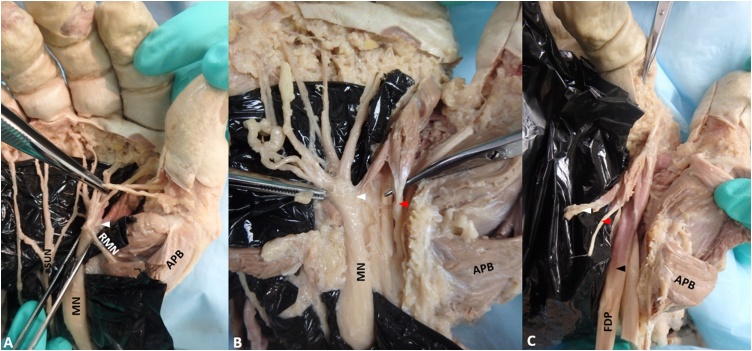
(A) SUN: Superficial branch of the Ulnar Nerve; MN: Median Nerve; RMN: Recurrent branch of the Median Nerve; APB: Abductor Pollicis Brevis; White Arrowhead: Belly of first lumbrical originating from the epineurium of the median nerve; (B) Red Arrowhead: Belly of first lumbrical originating from the flexor tendon sheaths; (C) Black Arrowhead: Belly of first lumbrical originating from the FDP: Flexor Digitorum Profundus tendon.

Further dissection reveal the first lumbrical muscle contained three unique heads. One aberrant belly originated from the epineurium of the median nerve (Fig. 4A). Another aberrant belly originated from the flexor tendon sheath within the carpal tunnel (Fig. 4B). The tendon of flexor digitorum superficialis passed between these two aberrant first lumbrical heads. The third/normal belly originated from the radial side of the flexor digitorum profundus tendon deep to the other two heads (Fig. 4C). All muscle bellies fused distally and inserted into the extensor expansion (Fig. 4C). To our knowledge, this is the first case of a tripennate first lumbrical muscle.

## 3 Discussion and conclusions

The hand described in this case study demonstrates an extranumerary muscle (FDML), tripennate lumbrical and the anomalous innervation of the FDMB muscle combined with several common and rare anatomical variations of nerves. The additional flexor muscle found did not match any previous descriptions. Though there have been prior reports of a flexor digiti minimi longus (FDML), none report of its muscle belly passing through Guyon’s canal. Due to the space-occupying nature of this variant FDML, the authors hypothesize that compression may have occurred within Guyon’s canal; evidence of this is provided by the tortuous ulnar artery. Compression by an anomalous muscle should be on the differential when a patient with unlikely demographics suffers from median and ulnar nerve compression. In addition to the presence of FDML, an anomaly of the first lumbrical was observed. There has been no report of a variant first lumbrical muscle with three origins. While it is not clear if muscle function was affected, the two aberrant origins may have contributed to compression of the median nerve. Lastly, the study describes a Berretini anastomosis, a relatively rare finding Kaplan’s anastomosis, and an innervation of flexor digiti minimi brevis by branches of the superficial ulnar nerve, not previously reported. This combination of unique nervous anastomoses may have confounded the clinical presentation.

The numerous anatomical variations described in this case study call for extra care in surgical interventions. Without previous knowledge of such variations, these anomalous structures are prone to iatrogenic injuries during surgeries. As such, surgeons should be aware of different anomalies to avoid injuries when performing surgeries of the hand.

## Sources of funding

This research did not receive any specific grant from funding agencies in the public, commercial, or not-for-profit sectors.

## Ethical approval

This cadaveric study utilized donors that willed their bodies to the Body Donation Program at UT Health San Antonio for education and research. UTHSCSA Office of IRB deemed the project to be non-regulated research (IRB Protocol#:HSC20190552N).

## Consent

The body donor used in this study was donated to the Body Donation Program at UT Health San Antonio for education and research. UT Health San Antonio is a member institution of the State Anatomical Board of Texas. All donations to the program are regulated and cared for under the statutes and guidelines of the State of Texas.

## Author’s contribution

HLN, APO, and SYJ, dissected the right forearm and hand in the cadaver, obtained photographs, and labeled these images. SYJ, SWJ, APO, and HLN were major contributors in the writing of the manuscript. All authors read and approved the final manuscript.

## Registration of research studies

N/A.

## Guarantor

Haley Nation Ph.D.

## Provenance and peer review

Editorially reviewed, not externally peer-reviewed.

## Availability of data and materials

Data sharing is not applicable to this article as no datasets were generated or analyzed during the current study.

## Authors’ information

HLN (corresponding author) and APO are Assistant Professors in the Department of Cell Systems in Anatomy at UTHSCSA. HLN earned her Ph.D. in Anatomy from Penn State University and APO is a board certified general and plastic surgeon earning her MD from UTHSCSA. SYJ and SWJ are current third year medical students at UTHSCSA.

## CRediT authorship contribution statement

**H.L. Nation:** Conceptualization, Data curation, Investigation, Methodology, Project administration, Supervision, Writing - review & editing. **S.Y. Jeong:** Data curation, Investigation, Methodology, Writing - original draft. **S.W. Jeong:** Investigation, Writing - original draft. **A.P. Occhialini:** Data curation, Investigation, Methodology, Writing - review & editing.

## Declaration of Competing Interest

The authors declare that they have no competing interests.
